# Aging Impairs the Ability of Conventional Dendritic Cells to Cross-Prime CD8^+^ T Cells upon Stimulation with a TLR7 Ligand

**DOI:** 10.1371/journal.pone.0140672

**Published:** 2015-10-16

**Authors:** Estefanía R. Zacca, María I. Crespo, Rachel P. Acland, Emiliano Roselli, Nicolás G. Núñez, Mariana Maccioni, Belkys A. Maletto, María C. Pistoresi-Palencia, Gabriel Morón

**Affiliations:** Centro de Investigaciones en Bioquímica Clínica e Inmunología, Consejo Nacional de Investigaciones Científicas y Técnicas, Facultad de Ciencias Químicas, Universidad Nacional de Córdoba, Córdoba, Argentina; National Council of Sciences (CONICET), ARGENTINA

## Abstract

The aging process is accompanied by altered immune system functioning and an increased risk of infection. Dendritic cells (DCs) are antigen-presenting cells that play a key role in both adaptive and innate immunity, but how aging affects DCs and their influence on immunity has not been thoroughly established. Here we examined the function of conventional DCs (cDCs) in old mice after TLR7 stimulation, focusing on their ability to cross-prime CD8^+^ T cells. Using polyU, a synthetic ssRNA analog, as TLR7 ligand and OVA as an antigen (Ag) model, we found that cDCs from old mice have a poor ability to stimulate a CD8^+^ T cell-mediated cytotoxic response. cDCs from old mice exhibit alterations in Ag-processing machinery and TLR7 activation. Remarkably, CD8α^+^ cDCs from old mice have an impaired ability to activate naïve CD8^+^ T cells and, moreover, a lower capacity to mature and to process exogenous Ag. Taken together, our results suggest that immunosenescence impacts cDC function, affecting the activation of naïve CD8^+^ T cells and the generation of effector cytotoxic T cells.

## Introduction

Aging impacts the homeostatic function of many systems, including the immune system, leading to a reduced ability to mount a robust immune response, in a process called immunosenescence [1–3]. Such changes in immune function result in increased susceptibility to and severity of viral and bacterial infections, increased incidence of cancer and autoimmune diseases, and a poor response to vaccination in the elderly [4–9].

Reduced immune responsiveness has been mainly attributed to alterations in the output and functions of lymphocytes, which are the primary mediators of adaptive immunity [10]. However, other cells of the immune system may also be playing a role in immunosenescense. In particular, dendritic cells (DCs) are Ag-presenting cells (APCs) that play a key role in mediating both adaptive and innate immunity [11]. DCs capture and process Ags for T cell priming, and can rapidly differentiate and mature in response to various stimuli, to produce pro- or anti-inflammatory cytokines that influence the outcome of the immune response. Among different DC subsets, CD8α^+^ conventional DCs (CD8α^+^ cDCs) are particularly suited for a process known as Ag cross-presentation, that is, for presenting exogenous non-cytosolic Ag bound to MHC I molecules to CD8^+^ T cells [12,13]. This process is essential to cross-prime CTLs, by which DCs activate naïve cytotoxic CD8^+^ T cells specific for Ag not expressed in DCs, such as pathogens unable to infect them, as well as tumor or dead cells [14]. Considering the fundamental role of DCs at the crossroads between the innate and adaptive immune response, it is conceivable that DCs may be partially involved in the alterations observed in T cell responses during aging. Recent studies on this subject do not reach a consensus about the functional status of DCs during aging [5–7,15–17]. In this study, we examined the effects of aging on the ability of cDCs to cross-present exogenous Ag to CD8^+^ T cells and to induce cross-priming after TLR7 stimulation. We found age-related defects in cDC cross-presentation machinery and cDC activation that contribute to impaired CD8^+^ T cell cross-priming. These may have a negative impact on the generation of a robust CD8^+^ T cell cytotoxic immune response in the elderly.

## Results

### cDCs from old mice have an impaired ability to stimulate a CD8^+^ T cell-mediated cytotoxic response

**Fig 1:**
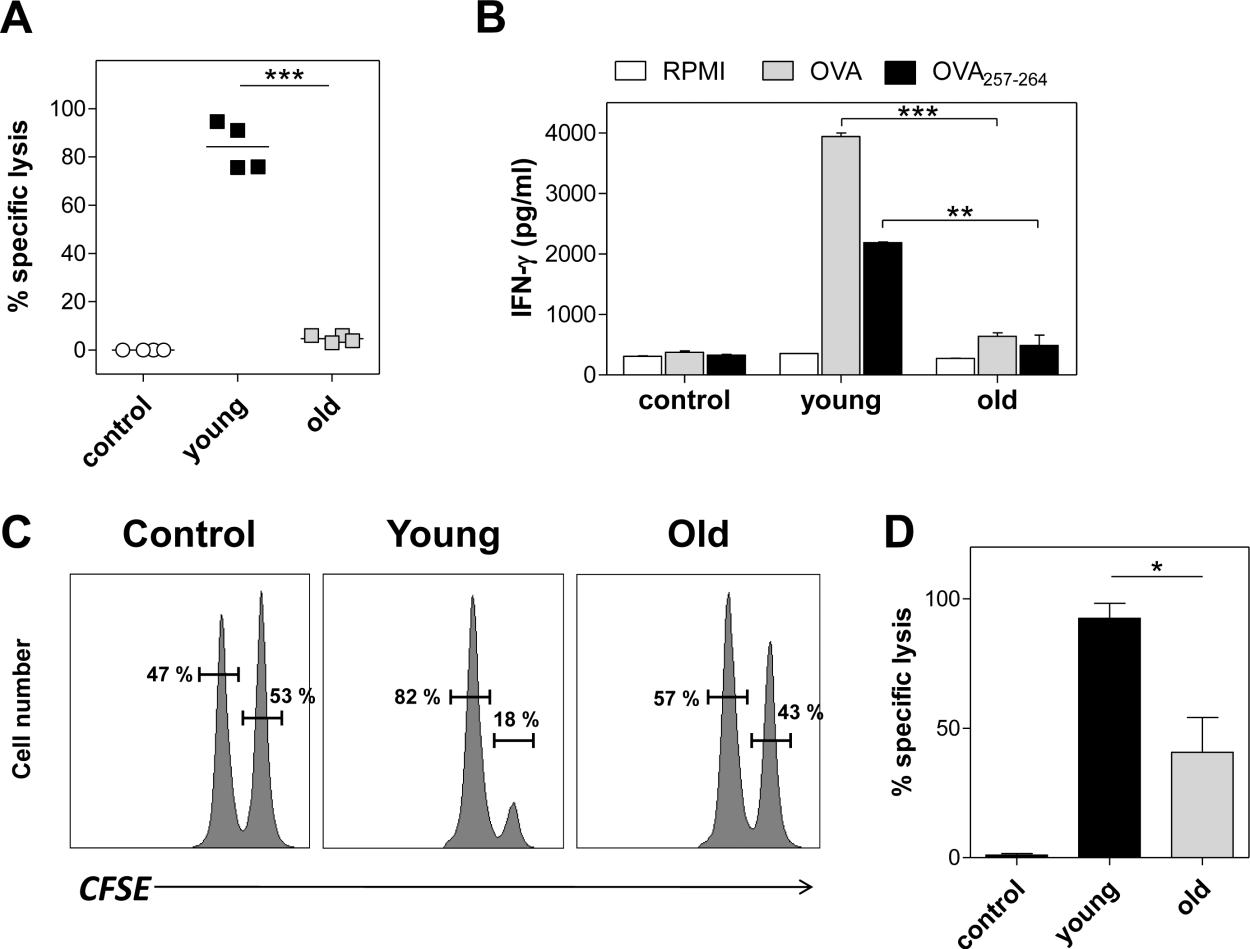
Effect of aging on the induction of CTL response. (A and B) Young and old C57BL/6 mice were immunized with a single intravenous injection of 2.5x10^9^ OVA-beads in 100 μg polyU/DO. Additional young and old mice were injected with saline as control. Seven days later, CTL response was determined by an *in vivo* killing assay. (A) Data show the percentage of specific *in vivo* killing of each individual mouse and the bars indicate the mean of each group. (B) IFN-γ content in culture supernatants of splenocytes from immunized mice determined by ELISA. Spleen cells were recovered and cultured for 72 hours in the presence of OVA or OVA_257–264_. (C and D) cDCs purified from the spleen of young and old C57BL/6 mice were incubated with 20 mg/mL OVA in 20 μg/mL polyU/DO, or with RPMI alone (control) for 90 minutes and then washed twice. One million cDCs per age group were intravenously injected into young C57BL/6 mice. Seven days later, CTL was determined by *in vivo* killing assay. (C) Representative flow cytometry histograms gated on CFSE^+^ cells are shown. (D) Data show the percentage of specific *in vivo* killing values, expressed as mean ± SEM. *p < 0.05, **p < 0.01, ***p < 0.001. Results are representative of 3 independent experiments (4 mice/age group/experiment). In all cases, young and old control groups gave similar results, and only the results of the young control group are depicted.

We recently reported that polyU complexed with DOTAP (polyU/DO) is a potent inducer of cytotoxic immune responses in a TLR7-dependent fashion [18]. With this in mind, in this study we first evaluated whether aging affects an Ag-specific cytotoxic response induced by TLR7 stimulation with polyU/DO. We intravenously immunized young and old mice with ovalbumin (OVA) as a monitor Ag coated to polystyrene beads (OVA beads) plus polyU/DO. Seven days later, we determined the presence of CTLs by an in vivo killing assay. To this end, OVA_257–264_-pulsed naïve syngeneic splenocyte targets (CFSE^high^-labeled cells) were intravenously injected into immunized mice. As an internal control, equal numbers of nonpulsed naïve syngeneic splenocytes (CFSE^low^-labeled cells) were injected. The number of CFSE^+^ cells remaining in the spleen after 24 hours was determined by flow cytometry. Immunization with OVA beads plus polyU/DO led to a potent cytotoxic response in young mice, whereas old mice did not develop a CTL response against OVA (Fig 1A). In addition to Ag-specific killing, we also determined IFN-γ secretion in the supernatants of splenocytes of these groups after being restimulated *in vitro* with whole OVA or OVA_257–264_ during 72 hours. We found that splenocytes from young immunized mice secreted higher levels of IFN-γ than splenocytes from old immunized mice (Fig 1B).

DCs have been clearly recognized as being the only APC capable of stimulating naïve T cells for CTL response. To evaluate the contribution of DCs to the diminished CTL response observed in old mice, we transferred cDCs from young and old donors to young hosts. In this way, we excluded the effect of aging on CD8^+^ T cells by using only young mice as cDC recipients. cDCs were purified from the spleen of young and old mice and then were incubated with OVA plus polyU/DO or with RPMI alone before their transfer into young hosts. The viability of purified cDCs from young and old mice was always 90–95% as assessed by trypan blue dye exclusion. Seven days after intravenous injection, young mice receiving OVA plus polyU/DO-preincubated cDCs from young mice developed a strong and specific CTL response (Fig 1C and 1D). In contrast, young mice that received OVA plus polyU/DO-preincubated cDCs from old mice exhibited a lower percentage of specific lysis. No response was induced in mice that received unstimulated cDCs. These results suggest that cDCs from old mice are less effective to induce a cytotoxic response against OVA upon TLR7 stimulation in young hosts.

### cDCs from old mice have impaired ability to cross-prime naïve CD8^+^ T cells *in vitro*

Generation of CTL responses requires priming of naïve CD8^+^ T cells by DCs. The fate of naïve T cells is determined by three signals that are provided by mature DCs [19]. Signal 1 is given by TCR-peptide-MHC interactions. Signal 2 comprises T-cell co-stimulation through molecules expressed on DCs such as CD40, CD80 and CD86. Finally, signal 3 is provided by polarizing cytokines secreted by DCs and other cell types, which determines the fate of the immune response [20]. The three signals are necessary to achieve optimal clonal expansion and the development of effector function [21].

**Fig 2:**
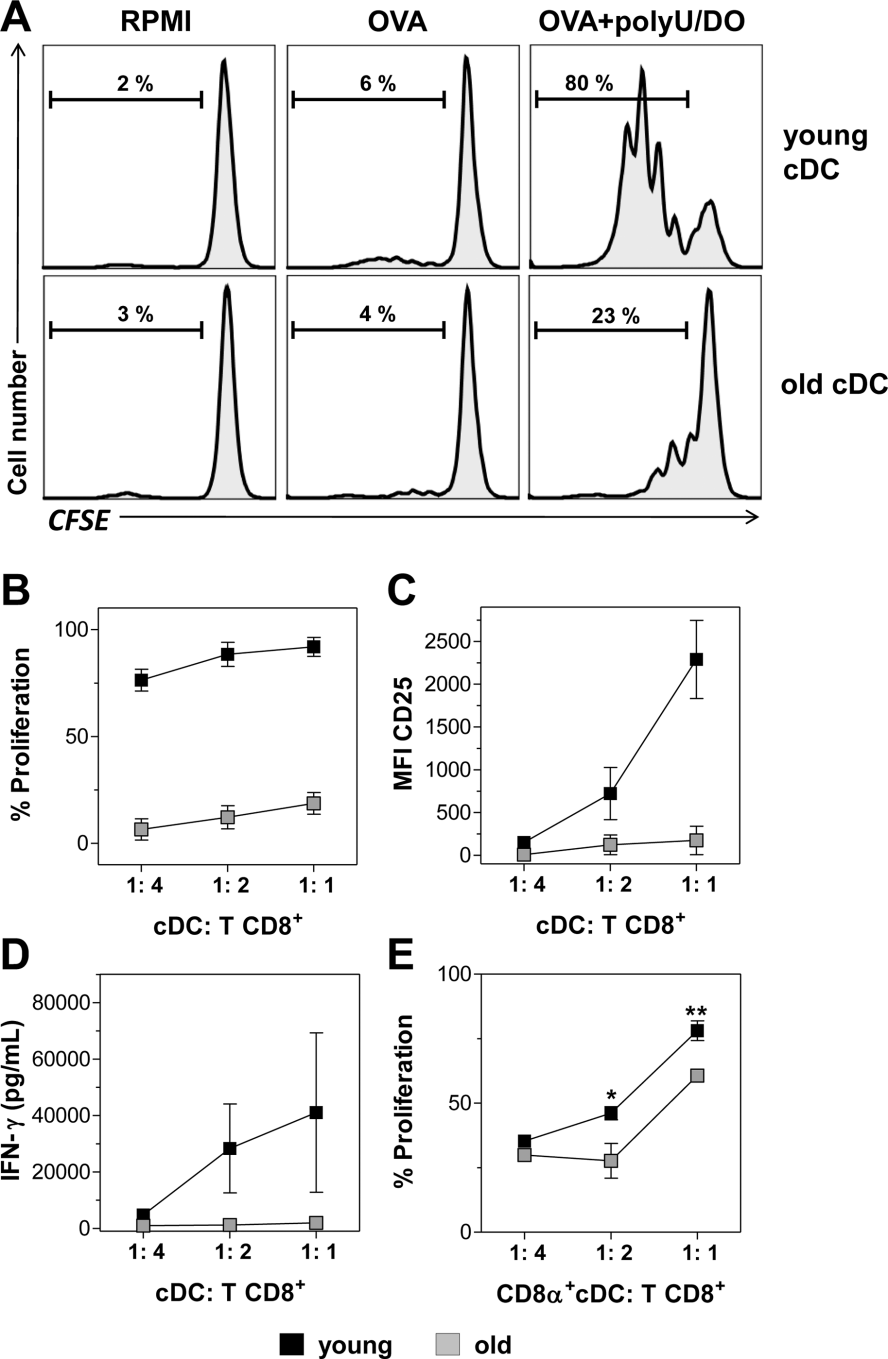
Aged splenic cDCs have impaired ability to cross-prime naïve CD8^+^ T cells *in vitro*. Total (A-D) or CD8α^+^ (E) cDCs purified from young and old mice were incubated with 1 mg/mL OVA mixed with 20 μg/mL polyU/DO for 90 minutes. Additional cDCs from young and old mice were incubated with RPMI or OVA as control. cDCs were then washed and cultured for 3 days with CFSE-labeled CD8β^+^ T cells isolated from the spleen of OT-I mice at different DC:T cell ratios. After culture, T cell proliferation and CD25 expression were analyzed by flow cytometry. (A) Representative histograms of T cell proliferation are shown from 1:1 ratio. (B, E) Percentages of proliferating T cells, (C) CD25 expression and (D) IFN-γ content in culture supernatants, determined by ELISA. Data show the mean ± SEM. *p < 0.05, **p < 0.01. Results are representative of 3 independent experiments (3–4 mice/age group/experiment).

In order to examine the effect of aged DCs in this important interaction, we first compared the ability of cDCs from young and from old mice to cross-prime naïve CD8^+^ T cells *in vitro*. Spleen cDCs from young or old mice were preincubated with OVA plus polyU/DO and cultured with CFSE-labeled CD8^+^ T cells isolated from young OT-I mice. Proliferation of CD8^+^ T cells was determined by the dilution of CFSE content in CD3^+^ 7-AAD^-^ cells and their activation by the expression of IL-2β-chain receptor (CD25) and IFN-γ secretion. As shown in Fig 2A and 2B, a high percentage of CD8^+^ T cells proliferated and upregulated CD25 expression (Fig 2C) in the presence of cDCs from young mice stimulated with OVA plus polyU/DO. In contrast, CD8^+^ T cells incubated with cDCs from old mice stimulated with OVA plus polyU/DO proliferated poorly (Fig 2A and 2B) and failed to upregulate CD25 expression (Fig 2C). cDCs from young and from old mice incubated with RPMI alone or with OVA alone, neither activated CD8^+^ T cell proliferation (Fig 2A) nor increased CD25 expression (data not shown). Moreover, CD8^+^ T cells cultured with cDCs from young mice actively secreted IFN-γ, whereas those cultured with cDCs from old mice secreted low or negligible IFN-γ (Fig 2D). CD8^+^ T cells culture with cDCs from young and from old mice incubated with RPMI alone or with OVA alone showed undetectable levels of IFN-γ (data not shown). Altogether, these results indicate that cDCs from old mice were less efficient to cross-prime naïve CD8^+^ T cells than cDCs from young mice.

Three DC subpopulations are present in murine spleen: CD8α^-^ or CD8α^+^ CD11c^high^ cDCs [12,22] and PDCA-1^+^ B220^+^ CD11c^int^ plasmacytoid DCs (pDCs) [23]. Among DC subsets, many different experimental systems have revealed that CD8α^+^ lymphoid-organ-resident cDCs are the most efficient cells cross-presenting Ags to CD8^+^ T cells, followed by migratory CD103^+^ DCs and inflammatory DCs [24–27]. Consistent with this data, we recently reported that in our experimental conditions, upon *in vitro* stimulation of sorted DC subsets with polyU/DO plus OVA, the CD8α^+^ cDCs were responsible for efficient CD8^+^ T cell proliferation [18]. When we evaluated CD8^+^ T cell proliferation induced by cDCs from young and from old mice, we used total cDCs, including both CD8α^+^ cDC and CD8α^-^ cDC (Fig 2A and 2B). As a lower percentage of the CD8α^+^ subset has been reported among cDCs in the spleen of old mice [5,16,28], we next asked whether the differences in CD8^+^ T cell cross-priming is a consequence of a lower percentage of the CD8α^+^ cDC subset or whether this reflects an inherent defect in CD8α^+^ cDC function, or both.

To address this, we performed an *in vitro* proliferation assay and evaluated the ability of purified CD8α^+^ cDCs to cross-prime CD8^+^ T cells. We found that CD8α^+^ cDCs from young mice stimulated with OVA plus polyU/DO induce a greater T cell proliferation than CD8α^+^ cDCs from old mice (Fig 2E and S1 Fig), indicating that the ability of CD8α^+^ cDCs to induce OVA-specific CD8^+^ T cell cross-priming is also impaired with aging. Again, CD8α^+^ cDCs from young and from old mice incubated with RPMI alone or with OVA alone did not activate CD8^+^ T cell proliferation (S1 Fig). Furthermore, we performed a characterization of spleen DC subset composition in young and old mice, in order to describe this in our experimental system. Representative dot plots with gating strategy from young mice are shown in S2A and S2C Fig. We found a significant decrease in CD8α^+^ cDC number and frequency in the spleen of old mice compared to the young ones (S2B Fig). pDC subsets decreased in frequency but not in number and the CD8α^-^ cDCs’ number and frequency were unaffected by aging. We also found a reduced frequency of total cDCs in the spleen of old mice compared to that of young mice (S2D Fig). However, we found no differences in cDC absolute numbers (S2D Fig).

We then compared DC viability between young and old mice in our experimental system to rule out the possibility that the observed impairment in CD8^+^ T cell cross-priming by DCs from old mice was the result of DC death. Using a fixable viability dye staining, we found no significant differences in viability between young or old cDCs incubated with polyU/DO after 24h of culture (S3A Fig). Although a significant number of cDCs from young mice died after 24 hours of polyU/DO stimulation, no significant differences in the viability of cDCs from old mice were found with or without stimulation. As shown in S3B Fig, when we analyzed CD8α^+^ cDC, similar results were found.

Together, these data suggest that, besides their reduced presence in the spleen, CD8α^+^ cDCs from old mice are less efficient to cross-prime OVA to CD8^+^ T cells. As was observed in Fig 2B, when total cDCs from old mice were used, CD8^+^ T cells proliferated poorly. This could be explained by the reduced CD8α^+^ fraction present in total cDCs. When equal amounts of purified CD8α^+^ cDCs from young and from old mice were used (Fig 2E), a significantly lower but substantial CD8^+^ T cell proliferation induced by CD8α^+^ cDCs from old mice was observed compared to the young ones.

### The Ag-processing machinery of cDCs is altered with aging

**Fig 3:**
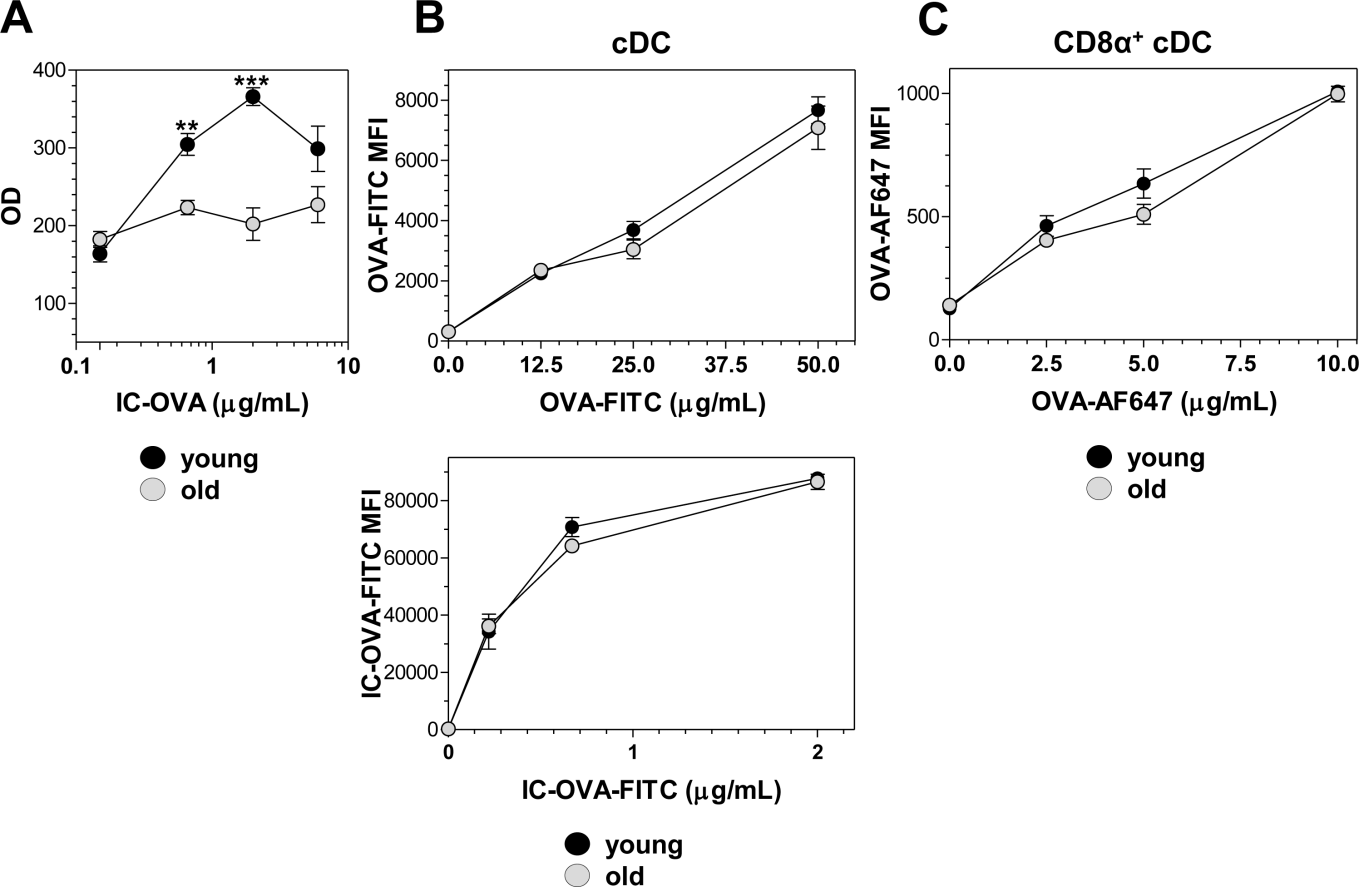
Ag presentation on MHC I molecules is affected in cDCs from old mice but Ag uptake is preserved. (A) *In vitro* Ag presentation assay. cDCs purified from the spleen of young and old mice were incubated with several OVA concentrations forming IC-OVA for 4 hours and then they were washed and incubated with B3Z cells overnight. B3Z stimulation, monitored by colorimetric bulk determination of β-galactosidase, is expressed as optical density (OD) at λ = 595. (B, C) *In vitro* Ag capture assay. (B) Spleen cells from young and old mice were recovered and incubated for 90 minutes with soluble OVA-FITC (upper) or IC-OVA-FITC (lower). Then, spleen cells were labeled with anti-CD11c Ab. Results are expressed as mean ± SEM of MFI in FITC channel. (C) CD8α^+^ cDCs purified from young and old mice were incubated for 90 minutes with soluble OVA-AF647. Results are expressed as mean ± SEM of MFI in AF647 channel. Results are representative of 3 independent experiments (3–4 mice/age group/experiment).

The initiation of an immunogenic CD8^+^ T cell response to an Ag that is not synthesized by the APC is known as cross-priming, and requires the ability of DCs to load peptides derived from exogenous Ags onto MHC class I molecules [29]. It is possible to determine the ability of DCs to present specific OVA_257-264_-K^b^ complexes on their cell surface by using an MHC I Ag presentation assay with the B3Z CD8^+^ T cell hybridoma, specific for the H2-K^b^–restricted OVA_257-264_ epitope. We performed an *in vitro* assay to assess the intrinsic ability of DCs (without any other influence than DCs themselves) to achieve MHC I Ag presentation. cDCs from young and old mice were incubated with different concentrations of OVA forming immune complexes (IC-OVA) and the presence of OVA_257-264_-K^b^ complexes on cDCs was monitored by the activation of B3Z cells (see Materials and Methods). As shown in Fig 3A, cDCs from young mice exhibited a greater ability to activate B3Z cells than cDCs from old mice. This indicates that aging decreased the ability of cDCs to cross-present the OVA_257-264_ peptide in MHC I molecules.

Activation of the B3Z CD8^+^ T cell hybridoma does not require co-stimulatory signaling, but requires active uptake and processing of OVA. We therefore investigated whether aging affects these mechanisms involved in DC Ag presentation. First, we examined the effect of aging on Ag uptake using soluble OVA protein coupled with FITC (OVA-FITC) or IC-OVA-FITC. *In vitro*, OVA-FITC uptake was equally efficient in young and old cDCs as no differences were found on the MFI in the FITC channel (Fig 3B, upper). A similar result was observed when spleen cells were incubated in the presence of different doses of OVA-FITC forming immune complexes (Fig 3B, lower). As CD8α^+^ cDCs’ ability to cross-prime CD8^+^ T cells *in vitro* is affected by aging (Fig 2E), we also performed experiments to dissect the mechanisms involved in this process in this cell subset. We carried on the uptake experiment with CD8α^+^ cDCs, using OVA coupled to pH-insensitive Alexa Fluor 647 (OVA-AF647). Again, no differences in Ag uptake were found between CD8α^+^ cDCs from young and old mice (Fig 3C).

**Fig 4:**
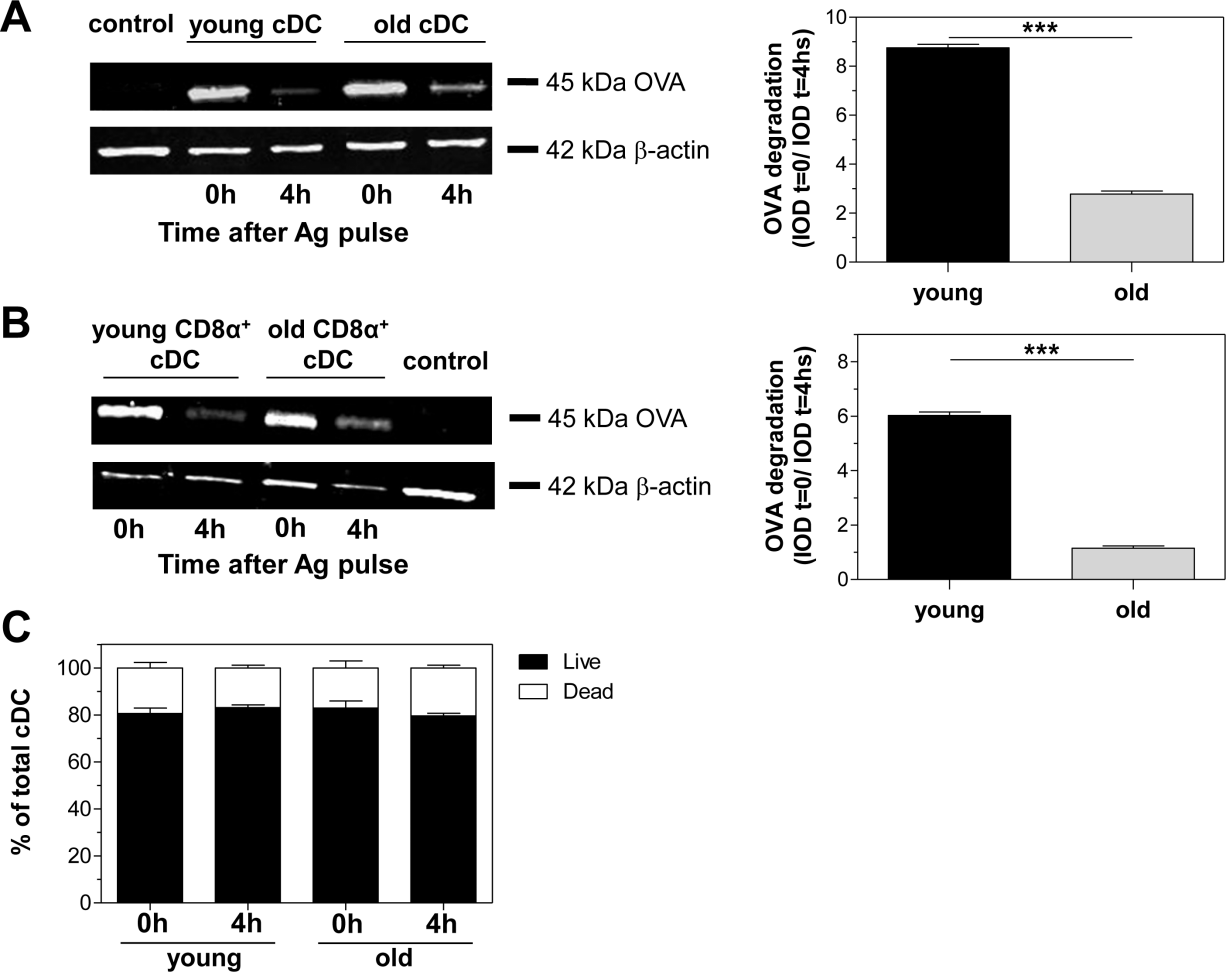
Ag degradation in cDCs is affected by aging. Persistence of OVA protein in cell lysates of total (A) or CD8α^+^ (B) cDCs purified from young and old mice was determined by Western blot after 1 hour pulse loading with 0.625 mg/mL OVA plus 20 μg/mL polyU/DO (time 0, 0h) and 4h chase. Actin was used for loading control. The control line contains total cell lysates of untouched splenic DCs. Densitometric analysis of Western blots (right) is expressed as the ratio of integrated optical density (IOD) at time 0 relative to IOD at chase time. (C) Percentages of total live and dead cDCs after 1 hour pulse loading and 4h chase. Data represent the mean ± SEM of duplicate cultures and are representative of 2 independent experiments. ***p < 0.001.

Considering that cDCs from old mice could efficiently internalize OVA, but poorly cross-present OVA to CD8^+^ T cells, we then analyzed OVA persistence in whole cell lysates of splenic cDCs from young and old mice by Western blot as an approach to assessing Ag processing. After a 60-minute pulse of cDCs with OVA plus polyU/DO (0 hour), cDC lysates from both young and old mice showed an equivalent 45 kDa OVA band (Fig 4A, left). Four hours later, the 45 kDa OVA band had almost completely disappeared in cDC lysates from young mice, while cDC lysates from old mice still showed a consistent band. Under the same conditions, CD8α^+^ cDCs from old mice showed a similar result to that using total cDCs (Fig 4B). As shown by densitometric analysis, neither total nor CD8α^+^ cDCs from old mice could degrade OVA as cDCs from young mice did at the times assayed (Fig 4A and 4B right). The OVA band detected in our plots is the result of an active uptake mechanism, and not only membrane-bound OVA. OVA uptake did not occur when performed at 4°C (data not shown). We also assayed the viability of cDCs under these Ag processing experimental conditions. Using 7AAD staining, we found no significant difference in viability between young or old cDCs after stimulation (Fig 4C). Ag persistence in cDCs from old mice was not due to a decrease in their viability. Together, these results clearly demonstrate that aging altered the ability of cDCs to process exogenous Ag, which would correlate with defects in Ag cross-presentation.

### DC maturation is affected by aging

**Fig 5:**
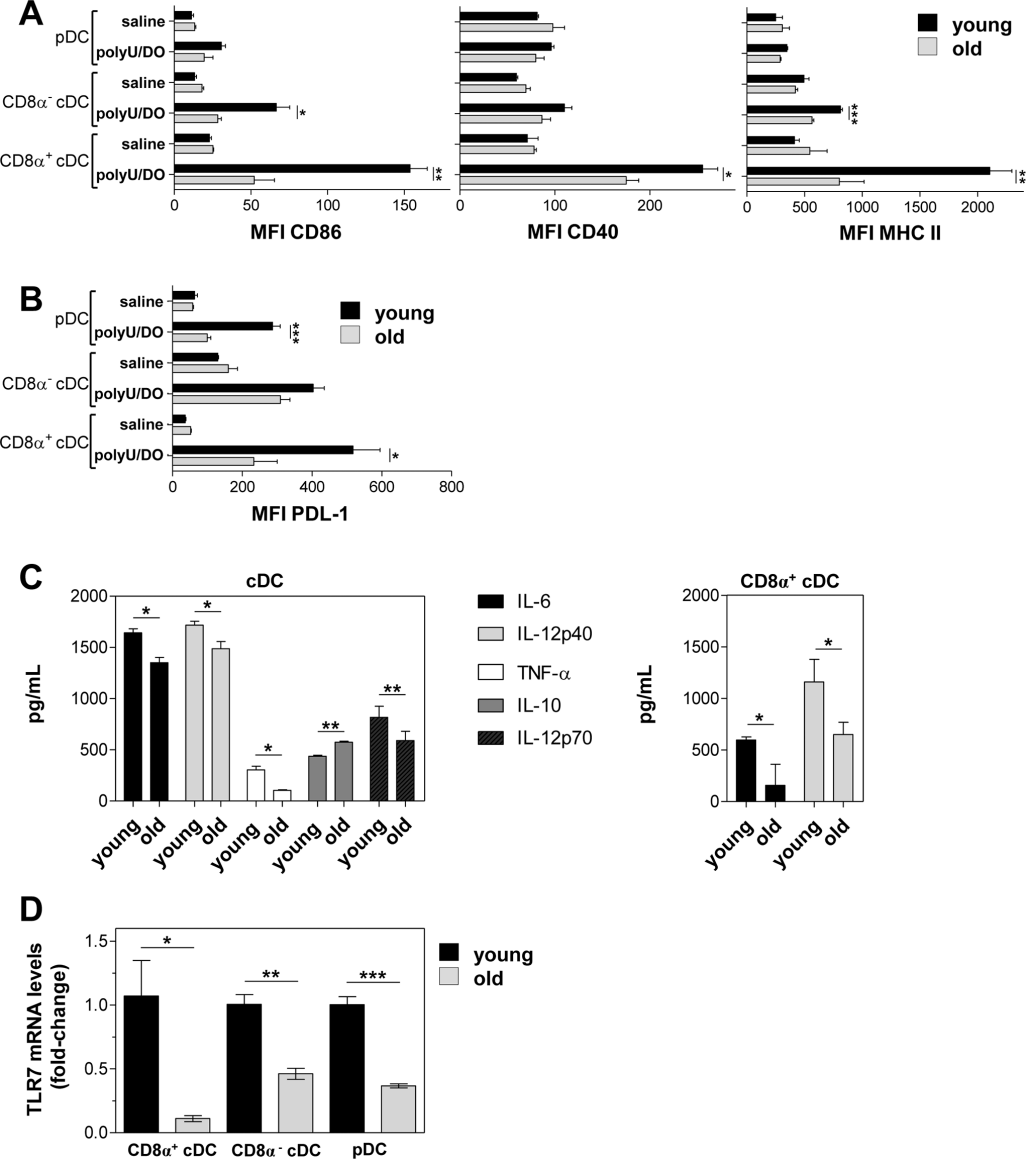
DC maturation is affected by aging. (A and B) Young and old mice were intravenously injected with 100 μg polyU/DO. Eighteen hours later, expression levels of CD86, CD40, MHC II (A) and PDL-1 (B) were determined in DC subsets by flow cytometry. Total (C) or CD8α^+^ (D) cDCs purified from the spleen of young and old mice were stimulated with 20 μg/mL polyU/DO and then supernatants were assayed for cytokine production by ELISA. (D) Spleen DC subsets from young and old mice were purified and total RNA of 1x10^6^ DCs was extracted. Relative mRNA levels for *Tlr7* were quantified by qPCR and normalized to *Hprt1*. Data show the mean ± SEM. Results are representative of 3 independent experiments (3–4 mice/age group/experiment). *p < 0.05, **p < 0.01, ***p < 0.001.

The outcome of T-cell responses depends on the DC maturation stage, as immature or semi-mature DCs have been found to induce T cell tolerance [30]. In order to compare the ability of DCs from young and old mice to mature upon TLR7 stimulation, we studied the upregulation of co-stimulatory molecules in splenic DC subsets. As shown in Fig 5A, the expression of all surface markers was not significantly different between DC subsets from young and old control mice. After intravenous injection with polyU/DO, cDCs from young mice upregulated CD86, CD40 and MHC II expression, whereas pDCs showed only a modest increase in CD86. In old mice injected with polyU/DO, CD8α^-^ cDC showed a lower upregulation of CD86 and MHC II than their young counterparts. Remarkably, CD8α^+^ cDC from old mice have a significantly lower upregulation of CD86, CD40 and MHC II than CD8α^+^ cDC from young mice. Furthermore, we evaluated PDL-1, a molecule that is involved in the inhibition of T and B cell responses, in order to rule out the possibility that the impairment in CD8^+^ T cell cross-priming by DCs from old mice was related to higher upregulation of PDL-1. After intravenous injection with polyU/DO, DC subsets from young and old mice upregulated PDL-1, although pDC and CD8α^+^ cDC showed a lower upregulation than their young counterparts (Fig 5B).

We then examined cytokine secretion in cDCs from old mice after *in vitro* polyU/DO stimulation. Supernatants of stimulated cDCs from old mice contained lower levels of TNF-α, IL-6, IL-12p40 and IL-12p70 and higher levels of IL-10 than supernatants of cDCs from young mice (Fig 5C, left). Notably, we observed mildly elevated levels of IL-6 in culture supernatants of cDCs from old mice without stimulus compared to those from young ones (137 ± 12 vs 76.4 ± 0.4 pg/mL respectively, p < 0.05), consistent with the pro-inflammatory milieu reported in older individuals [31]. When we examined cytokine secretion in culture supernatants of CD8α^+^ cDCs after polyU/DO stimulation we found that CD8α^+^ cDCs from old mice secreted lower levels of IL-6 and IL-12p40 than CD8α^+^ cDCs from young mice (Fig 5C, right).

It is now clear that TLR ligands not only stimulate transcription of cytokines and co-stimulatory molecules but also trigger an array of responses that affect the membrane vacuolar system, the cytoskeleton, and the machinery of protein translation and degradation [32]. Considering our results in which cDC maturation and cDC Ag-processing machinery were both affected by aging after polyU/DO stimulation, we next determined the relative TLR7 mRNA expression in DC subsets from old mice compared to their young counterparts. Very recently, TLR7 was appreciably detected on splenic pDCs and cDC subsets by ourselves and others [18,33]. By quantitative real time PCR, using *Hprt1* as a reference gene, we observed a reduced expression of *Tlr7* mRNA in all spleen DC subsets from old mice compared to those from young mice (Fig 5D). We repeated this quantification using *Gapdh* as a reference gene with similar results (data not shown).

Collectively, our results show that aging affects not only TCR-peptide-MHC interactions (signal 1) in cDCs, but also signals 2 and 3, because we observed reduced production of proinflammatory cytokines and lower upregulation of co-stimulatory molecules upon polyU/DO stimulation.

### Altered IκB-α phosphorylation in splenic cDCs from old mice

**Fig 6:**
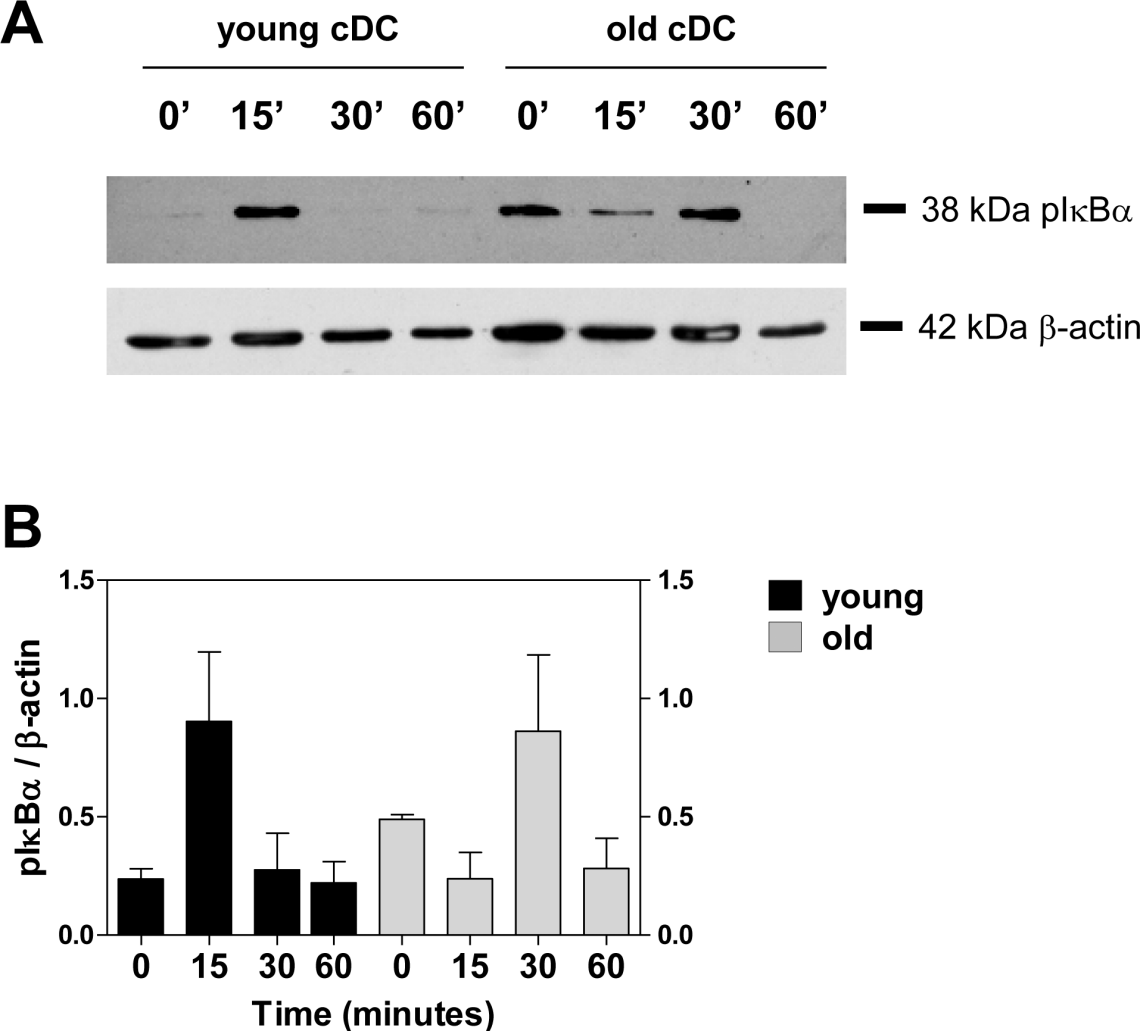
Differential phosphorylation of IκB-α in cDCs from young and old mice after TLR7 stimulation. (A) Pooled splenic cDC isolated from 3 young or old mice were incubated with 20 μg/mL polyU/DO for the indicated time. Cells were then lysed, and the level of phosphorylation of IκB-α was evaluated by Western blot using an anti-pIκB-α antibody. β-actin levels were used for loading control. (B) IOD of phosphorylated IκB-α normalized to β-actin. Data represent mean ± SEM of 2 pooled independent experiments.

NF-κB activation is crucial in TLR7-mediated DC maturation [34]. NF-κB activation via the canonical pathway is mediated by the upstream IκB kinase (IKK). Upon cellular stimulation, IKK is activated and phosphorylates IκB, which is then polyubiquitinated and degraded by proteasome. IκB degradation allows NF-κB to translocate to the nucleus, where it binds to its target sites [34,35]. As a read-out for NF-κB activation, we used Western blot to determine the phosphorylation of IκB-α in cDCs from young and old mice stimulated with polyU/DO. Unstimulated cDCs from young mice showed no phosphorylated IκB-α (pIκB-α), whereas 15 minutes after polyU/DO stimulation, cDCs from young mice showed a consistent pIκB-α band, which rapidly decreased to baseline values (Fig 6A and 6B). In contrast, unstimulated cDCs from old mice showed a strong band of pIκB-α. The presence of phosphorylated IκB-α in unstimulated cDCs from old mice was confirmed by the finding of nuclear immunoreactivity for the p65 subunit of NFκB (S4 Fig). Upon stimulation with polyU/DO, cDCs from old mice required 30 minutes of incubation to show an increase in pIκB-α level (Fig 6A and 6B), demonstrating an alteration in their downstream TLR7 signaling.

## Discussion

The DC is a critical player involved in regulating immunity and tolerance. Age-associated changes in DC development and function can significantly compromise the immune system and directly influence both adaptive and innate immunity in the elderly [6].

In this study, we examined the impact of aging on cDC function during TLR7 activation, focusing on their ability to cross-prime CD8^+^ T cells. Using polyU/DO, which activates DCs through TLR7, we found that cDCs from old mice exhibit an impaired ability to cross-prime naïve CD8^+^ T cells as compared to young cDCs. While some studies indicate an age-related defect in DC function [15,36–38], others report that DC function in aged individuals is fully preserved [16,39,40] or even enhanced [41]. Besides this, the effect of aging on cDC Ag cross-presentation has not been fully addressed until now. Intriguingly, we did not find age-associated differences in the ability of splenic cDCs stimulated with polyU/DO to activate CD4^+^ transgenic T cells *in vitro* (unpublished results). A similar result was observed by Wong et al. after stimulation with CpG [5].

For efficient Ag presentation and induction of a specific immune response by DCs, the number and stability of MHC I-peptide complexes are crucial. Our result showed that cDCs from old mice have a lower ability to cross-present OVA peptide/MHC I complexes to CD8^+^ T cells. As OVA uptake ability was similar between cDCs from young and old mice, the differences between their cDCs in Ag presentation and CD8^+^ T cell activation could be attributable to deficiencies in Ag processing machinery and cDC activation. In line with this possibility, CD8α^+^ cDCs from old mice presented a lower capacity to degrade OVA at early stages (Fig 5) compared to CD8α^+^ cDCs from young mice, although they finally degrade OVA over longer periods (24 hours, data not shown), similarly to CD8α^+^ cDCs from young mice. Furthermore, we observed that Ag persistence in cDCs from old mice was not due to a decrease in their viability. Ag cross-presentation is associated with lower Ag degradation in endosomes and enhanced Ag persistence [24]. However, in the present study we found lower Ag degradation and lower Ag cross-presentation. One important fact is that we evaluated OVA degradation in the whole DC, without differentiating endosomal and non-endosomal protein degradation, as endosomal studies require a huge number of DCs to be properly carried out. Thus, one possibility to consider is that either Ag translocation to cytosol or proteasomal degradation in aged DCs may be affected, impeding final OVA degradation in cytosol and thus preventing efficient Ag cross-presentation. Considering that it has been widely reported that the ubiquitin-proteasomal system is affected by aging [42,43], weak proteasomal degradation is a plausible hypothesis that needs to be confirmed in further studies.

CD8α^+^ cDCs are specialized at cross-presentation [44] and have developed specific adaptations of their endocytic pathway including high endosomal pH, low endosomal protein degradation, and high export to the cytosol [24]. We previously reported that stimulation of total and CD8α^+^ cDCs with polyU/DO leads to changes in their endocytic pathway that facilitate cross-presentation and cross-priming of CD8^+^ T cells [18]. Here we found that, besides their reduced presence in the spleen, CD8α^+^ cDCs from old mice are less efficient to cross-prime OVA to CD8^+^ T cells. These findings, among other alterations in different cells of the immune system, may be involved in the absent CTL response against OVA in old C57BL/6 mice. It is well known that CD4^+^ and CD8^+^ T cells responses are defective in older mice [10]. Furthermore, T helper cell activity through CD40-CD40 ligand interactions or proinflammatory cytokines are required for in vivo generation of CTLs, which we did not address in this study.

In addition to Ag cross-presentation to CD8^+^ T cells, we also observed an age-related reduction in proinflammatory cytokine production and a lower upregulation of co-stimulatory molecules by splenic DC subsets upon polyU/DO stimulation (Fig 5A and 5C). CD8α^+^ cDCs from old mice showed a markedly impaired ability to upregulate co-stimulatory molecules upon TLR7 stimulation (Fig 5C). Similar results were found by Li et al. [15], who found that CD8α^+^ cDCs from old mice show poor upregulation of co-stimulatory molecules at early time points following infection with *Listeria monocytogenes*. Our findings correlate with the lower levels of *Tlr7* mRNA observed in splenic DC subsets from old mice, suggesting that the lower activation of DC subsets after polyU/DO stimulation may be related at least in part to changes in TLR7 expression.

NF-κB has been proposed as the culprit of ‘‘inflammag-ing”, a low-grade, chronic, systemic inflammatory response associated with aging [45]. Consistent with this, we found a low but detectable level of phosphorylation of IκB-α in unstimulated cDCs from old mice compared to those from young ones, accompanied by the presence of the p65 subunit of NF-κB in their nucleus (Fig 6 and S4 Fig). Furthermore, we observed defects in IκB-α signaling in cDCs from old mice after TLR7 triggering (Fig 6A and 6B). It would be expected that these alterations in IκB-α signaling in addition to the lower levels on mRNA TLR7 in cDCs from old mice result in impaired maturation ability during aging. These results are in agreement with those of Panda et al., who found an age-associated decrease in TLR levels together with alterations in the functionality of primary human mDCs and pDCs [46].

Considering that TLR ligands have been noted as affecting Ag processing within the CD8^+^ cDCs and enhancing Ag cross-presentation [14,47], that polyU/DO stimulates efficient Ag cross-presentation responses in CD8α^+^ cDCs in young mice [18], and that CD8α^+^ cDCs from old mice are less efficient to cross-prime OVA to CD8^+^ T cells upon TLR7 ligation, it is possible that a defective response to TLR7 stimulation may be a key alteration in cDCs from old mice, notably in CD8α^+^ cDCs, without excluding that other aged-DC populations may contribute to the diminished cross-priming through interaction with CD8α^+^ cDCs or with CD8^+^ T cells.

As previously noted, the fate of naïve T cells is determined by three signals that are provided by mature DCs [19]. Clearly, our results show that aging affects these three signals in DCs, resulting in detrimental activation of naïve CD8^+^ T cells. Severe influenza virus infections are very common in the elderly [48,49], and the presence of an antiviral CD8^+^ T cell response has been associated with their protection against influenza [50]. TLR7 signaling plays an important role during influenza infection [51]. Furthermore, TLR7 plays a critical role in the induction of cross-protective immunity upon vaccination with whole inactivated influenza virus [52]. Our observations on deficient TLR7 signaling, together with impaired CD8^+^ T cell cross-priming by cDCs in old mice, provide new insights that need further investigation when designing strategies to improve the quality of current vaccination programs for the elderly.

## Materials and Methods

### Mice and cell lines

Six to 8-week-old (young) and 20 to 22-month old (old) female C57BL/6 mice were employed. OT-I T-cell receptor transgenic mice, specific for the K^b^-restricted ovalbumin 257–264 epitope (OVA_257-264_) [53] were provided by Dr. F.A. Goldbaum (Fundación Instituto Leloir, Buenos Aires, Argentina). Experiments were conducted with the approval of the Experimentation Animal Committee of the School of Chemical Sciences of the National University of Córdoba (authorization # 15-07-62010). Our animal facility meets the terms of the Guide to the Care and Use of Experimental Animals, published by the Canadian Council on Animal Care, and has the assurance number A5802-01 delivered by the Office of Laboratory Animal Welfare (National Institutes of Health). The mice were maintained under a standard light cycle (12 h light/dark) and were allowed free access to water and food. All animals were weighed and anesthetized with isofluorane. Mice were euthanized by CO2 inhalation or cervical dislocation before spleen tissue collection. B3Z, a CD8^+^ T cell hybridoma, specific for H2-K^b^ restricted OVA_257-264_ epitope [54], was a gift from Dr. N. Shastri (University of California, Berkeley, CA).

### Reagents

Chicken egg ovalbumin (OVA, Worthington Biochemical, Lakewood, NJ) was used in soluble form or as 2 types of particles, either forming immune complexes (IC-OVA) or covalently linked to synthetic polystyrene beads (OVA beads). IC-OVA were prepared incubating OVA/PBS at various concentrations with rabbit anti-OVA sera (Sigma-Aldrich). OVA beads were prepared as reported elsewhere [18] by covalently coupling 0.5 mg/mL OVA to 1 μm Polybead amino microspheres (Polysciences, Warrington, PA). OVA coupled to FITC or Alexa Fluor 647 (Molecular Probes, Invitrogen) and yellow-green (YG)-stained 1 μm polystyrene beads (fluorescent equivalent of OVA beads, Polysciences) were also employed. The peptide corresponding to the OVA_257-264_ epitope was synthesized by LANAIS-Pro (Buenos Aires, Argentina). PolyU/DO: 100 μg polyuridylic acid (polyU, Sigma-Aldrich, Buenos Aires, Argentina) was used as the TLR7 ligand, complexed to 60 μg DOTAP (Roche Diagnostics, Indianapolis, IN), in 20 mM HEPES-buffered saline (pH 7.4). Endotoxin content in polyU preparations, determined by a standard *Limulus* amebocyte lysate assay (BioWhittaker, Walkersville, MD) was less than 1 endotoxin unit/mL. Complete medium (CM) consisted of RPMI 1640 (Life Technologies Cell Culture Systems, Rockville, MD), supplemented with 1% L-Alanyl-L-Glutamine dipeptide (GlutaMAX I, Life Technologies), 10% FCS (Natocor), 5x10^-5^ M 2-mercaptoethanol (Sigma-Aldrich), and antibiotics (100 U/mL penicillin, 100 μg/mL streptomycin; PAA Laboratories, Pasching, Germany).

### Flow cytometry

Cells were co-incubated with fluorochrome-labeled antibodies (Abs) and anti-CD16/32 (clone 2.4G2) to block nonspecific binding to Fc receptors, for 20 minutes at 4°C. Cells were washed and 7-aminoactinomycin D (7-AAD) or Fixable Viability Dye (eBioscience, San Diego, CA) was then added to exclude dead cells. Monoclonal Abs (clone number) against the following Ags were employed: CD3 (145-2C11), CD4 (RM4-5), CD8 (53–6.7), CD8β (H35-17.2), CD11c (HL-3), CD86 (GL1), CD40 (HM40-3), I-A^b,d,q^/I-E^d,k^ (M5/114.15.2), CD25 (PC61.5), B220 (RA3-6B2), CD317 (PDCA-1, clone 129c) and Vβ 5.1, 5.2 TCR (MR9-4). A minimum of 1x10^5^ events were acquired on a FACSCanto II cytometer (BD Becton Dickinson Argentina, Buenos Aires, Argentina) and analyzed using FlowJo (Tree Star, Ashland, OR). All Abs were obtained from BD or eBioscience. Intensity of fluorescence signal is expressed as the geometric mean of the fluorescence intensity (MFI).

### Isolation of splenic DCs and CD8^+^ T cells

Splenic DCs were isolated as described in Morón et al. [55], after incubation of splenic cell suspensions with MACS-anti-CD11c (N418; Miltenyi Biotec, Bergisch Gladbach, Germany) and PE-Cy7-anti-CD11c (HL-3), selection on LS MACS columns (Miltenyi Biotec) and further sorting CD11c^high^ cDCs on a FACSAria IIu cell sorter (BD). In some experiments, cells were also labeled with anti-CD8α, anti-CD45R and anti-CD317 to separate DC subsets. CD8^+^ T cells were isolated from OT-I mice by incubation of spleen cells with anti-CD8β chain (H35-17.2) and further sorting on a FACSAria IIu. Purity of sorted cells was always > 98%.

### Cytokine detection assay

Cytokine levels were measured in culture supernatants by standard ELISA following instructions from the manufacturer (BD). The Ab pairs used were as follows (capture/biotinylated detection): IL-6, MP5-20F3/MP5-32C11; IL-12p40, C15.6/C17.8; IL-12p70, 9A5/C17.8; IFN-γ, R4-6A2/XMG1.2; TNF-α, 1F3F3D4/MP6-XT3/MP6-XT22; IL-10, JES5-2A5/JES5-16E3.

### *In vivo* killing assay

*In vivo* killing assay was performed as described in Morón et al. [55,56] using syngeneic splenocytes as target cells. Briefly, one group of syngeneic splenocytes (CFSE^high^) was pulsed with 10 μg/mL OVA_257–264_, and labeled with 3 μM CFSE (Molecular Probes). Another group of splenocytes (CFSE^low^) was labeled with 0.5 μM CFSE without peptide pulse as control. Then, equal numbers of CFSE^high^- and CFSE^low^-splenocytes were intravenously injected into mice. The number of CFSE^+^ cells remaining in the spleen after 24 hours was determined by flow cytometry. Cytotoxicity was expressed as the percentage of specific lysis, calculated from 100 x [1-(r_control_/r_immune_)], where r is given by the expression of % CFSE^low^/% CFSE^high^ cells for nonimmune (control) and immune mice, respectively. At the same time, 1.25x10^6^ splenocytes from the injected mice were incubated for 72 hours in the presence of 0.1 mg/mL OVA, 0.1 μg/mL OVA_257-264_ or CM as control. Supernatants were then collected and IFN-γ content was assayed by ELISA.

### T-cell proliferation assay

T-cell proliferation was assessed by coculturing splenic CD8β^+^ cells from OT-I mice with splenic DCs. Splenic CD8β^+^ cells from OT-I mice were previously stained with 5 μM CFSE in PBS 5% FCS and then extensively washed. Purified cDCs (0.5, 1 or 2x10^5^ cells/well) were incubated with 1mg/mL OVA mixed with 20 μg/mL polyU/DO at 37°C for 90 minutes in culture microplates in a final volume of 0.2 mL of CM. Then, DCs were washed and CFSE-labeled OT-I CD8^+^ T cells (2x10^5^ cells/well) were added. After 72 hours, the supernatants were collected for IFN-γ content assessment by ELISA and cultured cells were harvested, labeled with Abs against CD3 and CD25, and analyzed on a FACSCanto II flow cytometer. 7-AAD was added to samples before analysis to exclude dead cells. Proliferation was determined by the dilution of CFSE content in CD3^+^ 7AAD^-^ cells and is expressed as the percentage of cells under proliferation, that is, with a lower CFSE content than unstimulated cells at time 0 of culture.

### Ag presentation assay

Splenic cDCs (0.1 to 1 x 10^5^ cells/well) were pulsed with IC-OVA at 37°C for 4 hours in 96-well culture microplates. Then, cells were washed twice and incubated overnight at 37°C with 10^5^ B3Z cells/well. The stimulation of B3Z cells was monitored by colorimetric bulk determination of β-galactosidase activity in PBS-washed B3Z cells incubated for 4 hours with 0.15 mM chlorophenol red-β-D-galactopyranoside (CPRG, Roche Diagnostics Corporation) in 100 mM 2-mercaptoethanol, 9 mM MgCl_2_ and 0.125% NP40 detergent (IGEPAL CA 630, Sigma-Aldrich) in PBS [57].

### Quantitative RT-PCR

To analyze *Tlr7* mRNA expression, total RNA was extracted with TRIzol reagent from 1x10^6^ cells. Synthesis of cDNAs was primed with oligo(dT) followed by synthesis by Moloney murine leukemia virus reverse transcriptase (Promega). Quantitative RT-PCR was performed with 15 ng cDNA with SYBR Green PCR core reagents. The primers were: *Tlr7*, forward, 5′-GGATCTGCCATCCAGCTTAC-3′, reverse, 5′-ATTAGGTGGCAAAGTGGTGG-3′; *Hprt1*, forward, 5′-AAGCTTGCTGGTGAAAAGGA-3′, reverse, 5′-TCCAACAAAGTCTGGCCTGT-3′; *Gapdh*, forward, 5’-AGCCTCGTCCCGTAGACAA-3’, reverse 5’-AATCTCCACTTTGCCACTGC-3’. The condition cycling used was 95°C for 10 minutes followed by 40 cycles of denaturation at 95°C for 15 seconds and annealing for 1 minute at 60°C. To analyze the relative gene expression of *Tlr7* data, the 2(−ΔΔC_T_) method was used as previously described [58] and was relativized to the expression of the reference genes *Hprt1 and Gapdh*.

### Western blot analysis

Cells were washed in PBS to remove serum proteins, lysed in SDS sample buffer and boiled for 3 minutes at 100°C. For the OVA degradation experiment, cell lysates were separated on 10% polyacrylamide gel using SDS-PAGE and transferred to nitrocellulose membrane (Millipore, Billerica, USA). Blots were simultaneously incubated with rabbit polyclonal anti-OVA IgG (Natocor) and mouse anti-β-actin (as loading control). Signals were detected with anti-rabbit IgG IRDye 800CW and with anti-mouse IgG IRDye 680CW (LI-COR Biosciences, Lincoln, USA) using quantitative infrared fluorescence detection with *Odyssey* (LI-COR Biosciences). Blots were imaged in both the 700 and 800 nm channel by a single scan.

For IκB-α detection, cell lysates were separated on 15% polyacrylamide gel and transferred to nitrocellulose membranes. Blots were incubated with rabbit anti-pIκB-α (Ser32) primary antibody (Cell Signaling Technology, Inc., Danvers, MA). After incubation with HRP-conjugated goat anti-rabbit antibody (Cell Signaling), blots were revealed with ECL chemiluminescence. Membranes were then reprobed with mouse β-actin antibody for loading control and HRP-conjugated goat anti-mouse secondary antibody (Sigma-Aldrich).

### Fluorescence microscopy

Purified splenic DCs were fixed on slides after cytospin preparation. Cells were permeabilized and, after blockade, slides were stained with rabbit anti-p65 primary antibody (eBiosciences), Alexa Fluor 594-conjugated anti-rabbit IgG secondary antibody (Molecular Probes) and Hoescht 33258 (Molecular Probes) for nuclear staining. Finally, stained slides were mounted with Mowiol (Sigma-Aldrich) and analyzed using a Nikon TE2000-U microscope.

### Statistical analysis

Data were reported as mean ± SEM and were analyzed using GraphPad Prism software (GraphPad Software, San Diego, CA). *P* values were determined using the unpaired *t* test and two-way ANOVA followed by a Bonferroni´s posttest. Statistical significance was defined as *p* < 0.05.

## Supporting Information

S1 Fig Aged splenic CD8α^+^ cDCs have impaired ability to cross-prime naïve CD8^+^ T cells *in vitro*. CD8α^+^ cDCs purified from young and old mice were incubated with 1 mg/mL OVA mixed with 20 μg/mL polyU/DO for 90 minutes. Additional CD8α^+^ cDCs from young and old mice were incubated with RPMI or OVA as control. CD8α^+^ cDCs were then washed and cultured for 3 days with CFSE-labeled CD8β^+^ T cells isolated from the spleen of OT-I mice at different DC:T cell ratios. Representative histograms of T cell proliferation are shown from 1:1 ratio. Results are representative of 3 independent experiments (3–4 mice/age group/experiment).
(TIF)

S2 Fig DC content in young and old mice. (A) Representative dot plots with gating strategies analyzed by flow cytometry for spleen DC subsets from young mice are depicted. (B) Frequency and cell number of CD8α^+^ cDC (CD11c^high^ CD8α^+^), CD8α^-^ cDC (CD11c^high^ CD8α^-^) and pDC (CD11c^int^ B220^+^ PDCA-1^+^) present in the spleen from young and old C57BL/6 mice. (C) Representative dot plots with gating strategy for spleen cDC defined as CD11c^high^ from young mice. (D) Frequency and cell number of cDCs in spleens from young and old mice. Values are expressed as mean ± SEM. *p < 0.05, **p < 0.01, ns (no significant differences). Results are representative of 3 independent experiments (4 mice/age group/experiment).
(TIF)

S3 Fig Viability of DC from young and old mice after 24h of culture. Total (A) or CD8α^+^ (B) cDCs purified from young and old mice were incubated with 20 μg/mL polyU/DO or RPMI for 24h and then were stained with a fixable viability dye. (B) Percentages of total live and dead cells are shown. Values are expressed as mean ± SEM. **p < 0.01, ns (no significant differences) indicates statistical analysis between % of live cells per group. Results are representative of 3 independent experiments (4 mice/age group/experiment).
(TIF)

S4 Fig Basal NF-κB activation in cDCs from old mice. Spleen cDCs from young and old mice were purified and then fixed in slides after cytospin preparation without stimulus. Immunoreactivity of the p65 subunit of NF-kB (red) in cDCs was determined by confocal immunofluorescence. Hoescht labeling was used to visualize the nucleus (blue). Representative images of 3 independent experiments are shown (4 mice/age group/experiment).
(TIF)
