# Supplementary figures and images for: Aging Impairs the Ability of Conventional Dendritic Cells to Cross-Prime CD8+ T Cells upon Stimulation with a TLR7 Ligand
Source: PLoS One. 2015 Oct 16;10(10):e0140672. doi: 10.1371/journal.pone.0140672 (PMC4608578; doi:10.1371/journal.pone.0140672)

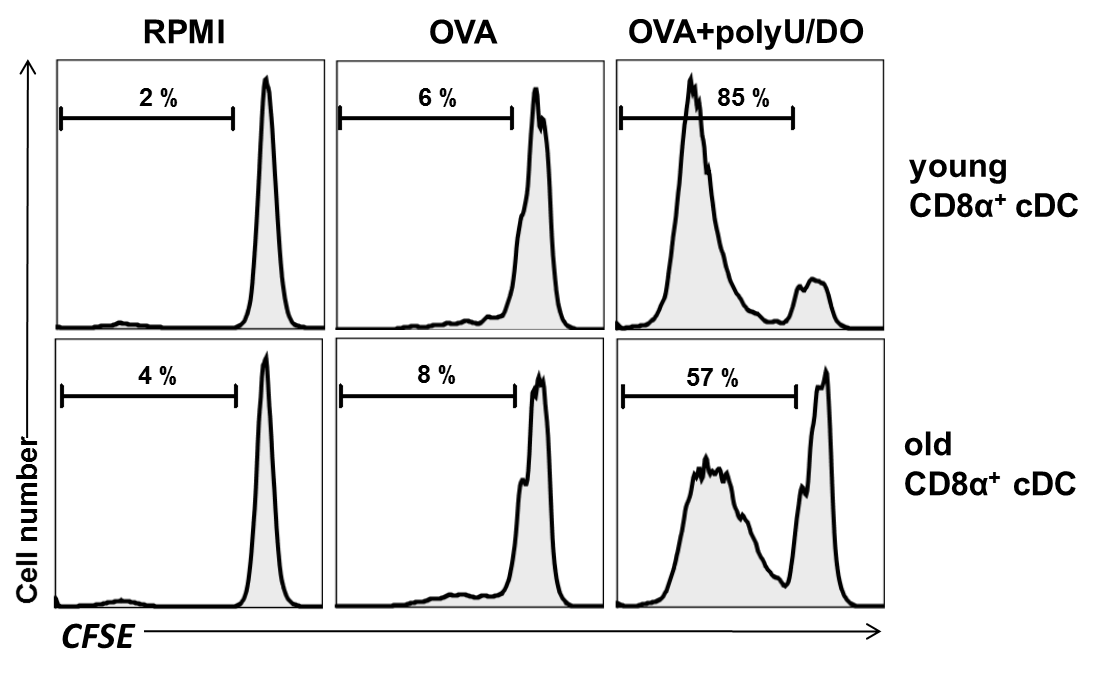

Supplement: S1 Fig — CD8α+ cDCs purified from young and old mice were incubated with 1 mg/mL OVA mixed with 20 μg/mL polyU/DO for 90 minutes. Additional CD8α+ cDCs from young and old mice were incubated with RPMI or OVA as control. CD8α+ cDCs were then washed and cultured for 3 days with CFSE-labeled CD8β+ T cells isolated from the spleen of OT-I mice at different DC:T cell ratios. Representative histograms of T cell proliferation are shown from 1:1 ratio. Results are representative of 3 independent experiments (3–4 mice/age group/experiment). (TIF) [file pone.0140672.s001.tif]

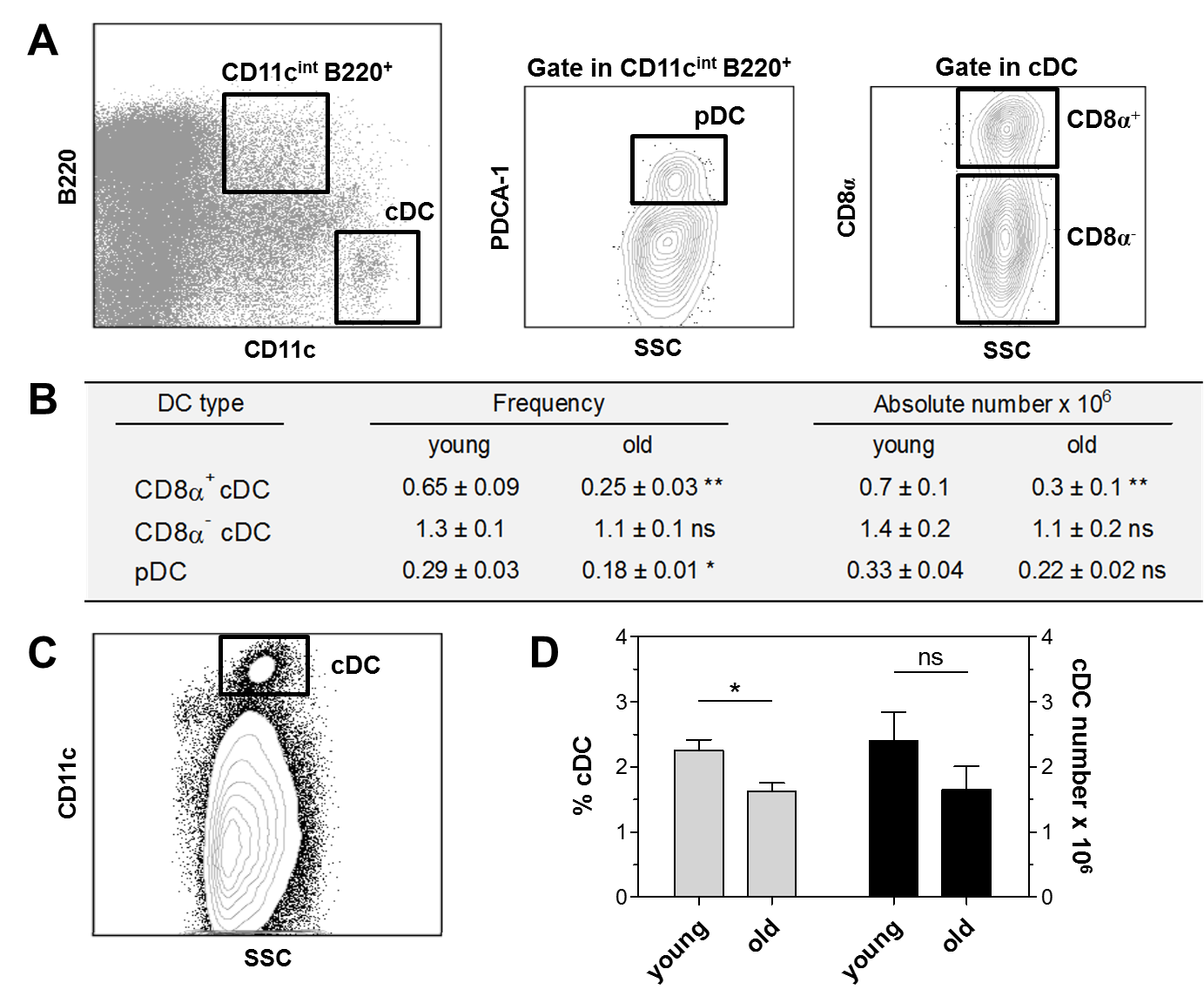

Supplement: S2 Fig — (A) Representative dot plots with gating strategies analyzed by flow cytometry for spleen DC subsets from young mice are depicted. (B) Frequency and cell number of CD8α+ cDC (CD11chigh CD8α+), CD8α- cDC (CD11chigh CD8α-) and pDC (CD11cint B220+ PDCA-1+) present in the spleen from young and old C57BL/6 mice. (C) Representative dot plots with gating strategy for spleen cDC defined as CD11chigh from young mice. (D) Frequency and cell number of cDCs in spleens from young and old mice. Values are expressed as mean ± SEM. *p < 0.05, **p < 0.01, ns (no significant differences). Results are representative of 3 independent experiments (4 mice/age group/experiment). (TIF) [file pone.0140672.s002.tif]

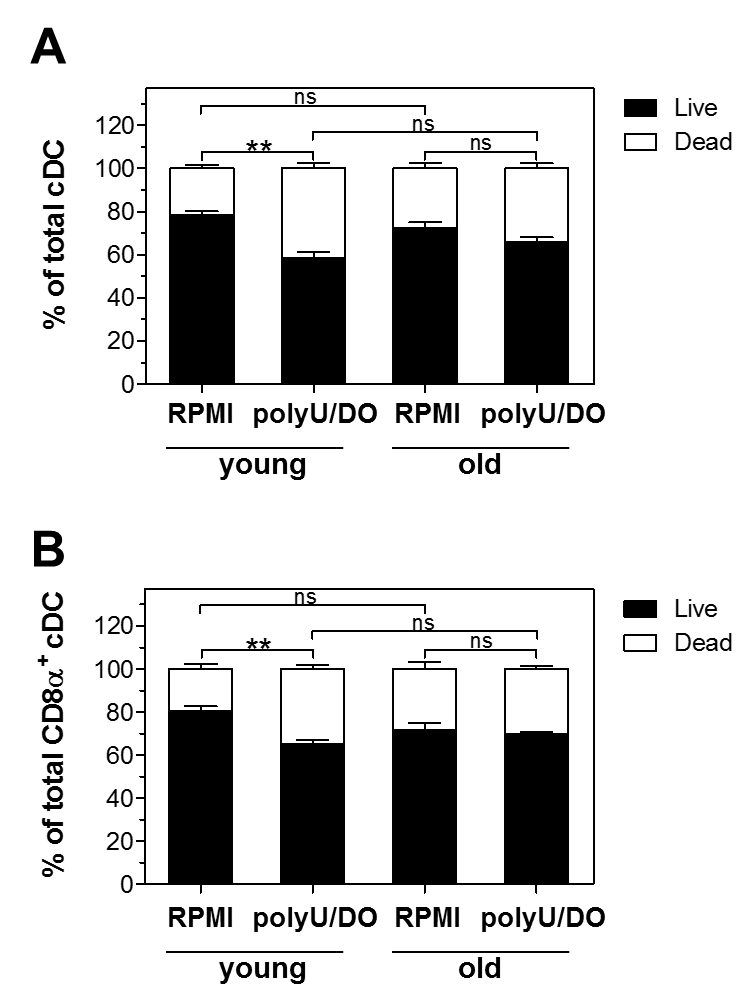

Supplement: S3 Fig — Total (A) or CD8α+ (B) cDCs purified from young and old mice were incubated with 20 μg/mL polyU/DO or RPMI for 24h and then were stained with a fixable viability dye. (B) Percentages of total live and dead cells are shown. Values are expressed as mean ± SEM. **p < 0.01, ns (no significant differences) indicates statistical analysis between % of live cells per group. Results are representative of 3 independent experiments (4 mice/age group/experiment). (TIF) [file pone.0140672.s003.tif]

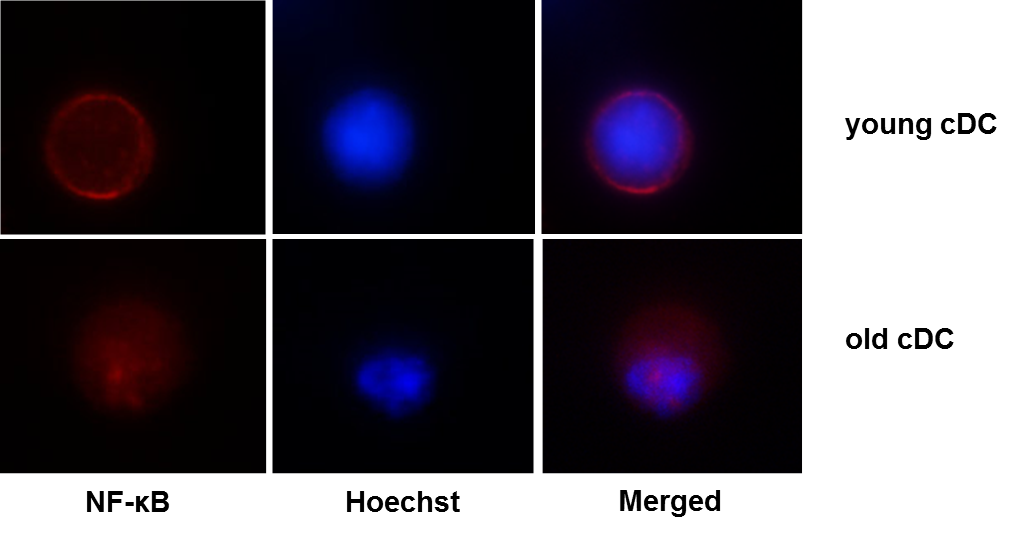

Supplement: S4 Fig — Spleen cDCs from young and old mice were purified and then fixed in slides after cytospin preparation without stimulus. Immunoreactivity of the p65 subunit of NF-kB (red) in cDCs was determined by confocal immunofluorescence. Hoescht labeling was used to visualize the nucleus (blue). Representative images of 3 independent experiments are shown (4 mice/age group/experiment). (TIF) [file pone.0140672.s004.tif]
